# Hesperidin Methyl Chalcone Reduces the Arthritis Caused by TiO_2_ in Mice: Targeting Inflammation, Oxidative Stress, Cytokine Production, and Nociceptor Sensory Neuron Activation

**DOI:** 10.3390/molecules28020872

**Published:** 2023-01-15

**Authors:** Nayara A. Artero, Marília F. Manchope, Thacyana T. Carvalho, Telma Saraiva-Santos, Mariana M. Bertozzi, Jessica A. Carneiro, Anelise Franciosi, Amanda M. Dionisio, Tiago H. Zaninelli, Victor Fattori, Camila R. Ferraz, Maiara Piva, Sandra S. Mizokami, Doumit Camilios-Neto, Rubia Casagrande, Waldiceu A. Verri

**Affiliations:** 1 Laboratory of Pain, Inflammation, Neuropathy and Cancer, Department of Pathology, Centre of Biological Sciences, Londrina State University, Londrina 86057-970, PR, Brazil; 2 Department of Biochemistry and Biotechnology, Centre of Exact Sciences, Londrina State University, Londrina 86057-970, PR, Brazil; 3 Department of Pharmaceutical Sciences, Centre of Health Science, Londrina State University, Londrina 86039-440, PR, Brazil

**Keywords:** inflammation, hesperidin methyl chalcone, titanium dioxide, prosthesis, arthritis, TRPV1, TRPA1, nociceptor sensory neuron, oxidative stress

## Abstract

Arthroplasty is an orthopedic surgical procedure that replaces a dysfunctional joint by an orthopedic prosthesis, thereby restoring joint function. Upon the use of the joint prosthesis, a wearing process begins, which releases components such as titanium dioxide (TiO_2_) that trigger an immune response in the periprosthetic tissue, leading to arthritis, arthroplasty failure, and the need for revision. Flavonoids belong to a class of natural polyphenolic compounds that possess antioxidant and anti-inflammatory activities. Hesperidin methyl chalcone’s (HMC) analgesic, anti-inflammatory, and antioxidant effects have been investigated in some models, but its activity against the arthritis caused by prosthesis-wearing molecules, such as TiO_2_, has not been investigated. Mice were treated with HMC (100 mg/kg, intraperitoneally (i.p.)) 24 h after intra-articular injection of 3 mg/joint of TiO_2_, which was used to induce chronic arthritis. HMC inhibited mechanical hyperalgesia, thermal hyperalgesia, joint edema, leukocyte recruitment, and oxidative stress in the knee joint (alterations in gp91^phox^, GSH, superoxide anion, and lipid peroxidation) and in recruited leukocytes (total reactive oxygen species and GSH); reduced patellar proteoglycan degradation; and decreased pro-inflammatory cytokine production. HMC also reduced the activation of nociceptor-sensory TRPV1^+^ and TRPA1^+^ neurons. These effects occurred without renal, hepatic, or gastric damage. Thus, HMC reduces arthritis triggered by TiO_2_, a component released upon wearing of prosthesis.

## 1. Introduction

Joint dysfunction can be induced by varied diseases, ranging from infections, chronic degeneration, tumors, and physical trauma [1]. The impairment of joint functionality and chronic pain drastically decrease mobility and the degree of engagement in common life interactions; thus, patients’ overall quality of life is affected. In severe cases, there is a need for arthroplasty, which is a surgical procedure ranging from the partial to total replacement of a dysfunctional joint by a prosthesis [2]. From January 2008 to December 2015, 47,289 total knee and total hip arthroplasties were performed in Brazil [3]. From 2006 to 2007, 62,196 hospitalizations for arthroplasty occurred in Canada, with an overall incidence of 81.2 cases per 100,000 individuals annually [4]. According to the Agency for Healthcare Research and Quality, in 2018, there were 715,200 knee arthroplasties performed in the United States [5]. It is expected that approximately 3.48 million arthroplasties will be performed by the year 2030 in the United States [6]. These data indicate that arthroplasty is an important surgical procedure that restores mobility, reduces pain, and improves overall quality of life [7]. Despite therapeutic success, the estimation of revision arthroplasty (the need for a prosthesis’s replacement) reaches nearly 10–15%, and, depending on the study, joint replacement failure can occur in 40% of cases [8]. Infection is an important cause of the need for revision arthroplasty; however, aseptic loosening is also common and occurs overtime due to the intrinsic wearing process that involves inflammation [2,9,10]. The aseptic loosening of a prosthesis may be accompanied by aggravating circumstances such as the increasing age of the patients and an increase in surgical risks; consequently, revision arthroplasty is not always possible [10]. Thus, the avoidance of the loosening of a prosthesis might be essential to prolong its use without the need for revision [11].

The wear of prostheses releases debris components that activate immune cells in periprosthetic tissue, leading to an aseptic inflammatory response [1]. Wear debris is rapidly phagocytosed by resident cells producing mediators such as tumor necrosis factor (TNF)α, interleukin (IL)-1β, IL-33, and reactive oxygen species (ROS) [12,13,14,15,16]. The production of such molecules explains the characteristic inflammation present in the wear of prostheses that involves the recruitment of inflammatory leukocytes, edema, cartilage degradation, and pain [15,16,17,18]. Titanium dioxide (TiO_2_) is a debris component released by the wear of a prosthesis. TiO_2_ is a white powder [19] commonly used in several products, such as additives in pharmaceuticals, dyes, sunscreen, and orthopedic prostheses [20]. Quite interestingly, when topically applied, nanosized TiO_2_ is absorbed through the skin, causing oxidative stress that affects the skin and even the liver [21]. Alongside the induction of skin inflammation by TiO_2_, it triggers reactive oxygen species (ROS) production by human epidermal cells [22]. In the lungs of mice, TiO_2_ triggers leukocyte recruitment with the production of chemokines, TNFα, and IL-1β [23]. In vitro, in microglia culture, TiO_2_ causes prolonged ROS production [24]. Considering that TiO_2_ is a prosthetic component, we were the first to explore its inflammatory characteristics in order to develop novel treatments targeting this specific type of arthritis [18]. We standardized a model of TiO_2_ articular inflammation, which is characterized by the recruitment of neutrophils and mononuclear cells, cartilage degradation, joint edema, and pain [18].

The flavonoid hesperidin methyl chalcone (HMC; 3,5,7-trihydroxy flavanone 7-rhamnoglucoside) is a current treatment for vascular diseases in a combination containing vitamin C and the extract of *Ruscus aculeatus* [25], which are commercialized under the commercial names Cyclo 3 Fort and BiCirkan [26]. We have shown that HMC reduces inflammation and pain in varied mouse models, including those triggered by formalin, capsaicin, carrageenan, complete Freund’s Adjuvant [27], zymosan [28], UVB irradiation of the skin [29,30], ulcerative colitis [31], and monosodium urate crystal-related gout arthritis [32]. Using an in silico approach together with confirmation in RAW 264.7 macrophages, we demonstrated that HMC reduces the activation of NFkB by reducing the phosphorylation of its Ser276 residue [28]. HMC also consistently reduced oxidative stress and cytokine production in those varied models [27,28,29,30,31]. Considering HMC’s mechanisms of action [27,28,29,30,31,32] and the pathophysiological mechanisms of articular inflammation caused by TiO_2_ [15], we reasoned that HMC could represent a potential therapeutic approach to the treatment of TiO_2_ arthritis; however, to our current knowledge, this hypothesis has not been investigated so far.

## 2. Results

### 2.1. Hesperidin Methyl Chalcone (HMC) Reduces Mechanical and Thermal Hyperalgesia and Weight Distribution Imbalance in the Titanium Dioxide (TiO_2_)-Induced Arthritis Model

**Figure 1:**
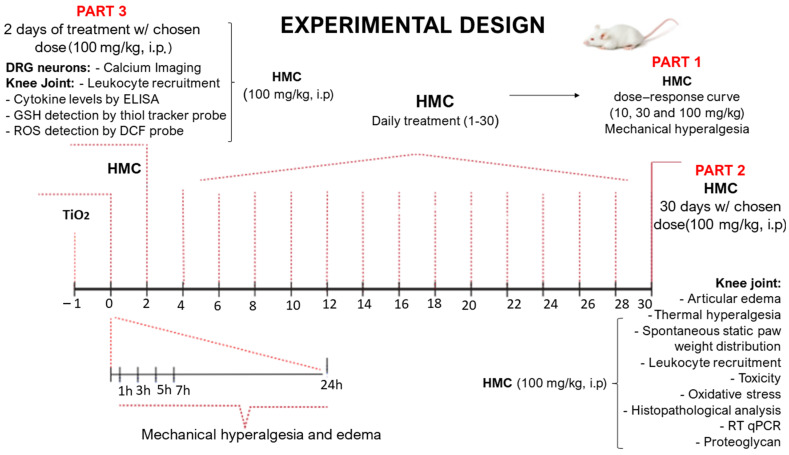
Schematic representation of hesperidin methyl chalcone (HMC) treatment protocol and titanium dioxide (TiO_2_) arthritis model. Part 1: mice received an intra-articular injection of TiO_2_ (3 mg/joint). HMC treatment (10, 30, and 100 mg/kg, administered i.p., diluted in saline) was initiated 24 h after TiO_2_ stimulus injection. Mechanical hyperalgesia was measured for up to 30 days (part 1). An HMC dose (100 mg/kg) was selected as per a dose–response curve shown in Figure 2A, and it was then used in all following experiments. Part 2: HMC activity against TiO_2_ arthritis was tested for 30 days or with sample collected at the 30th day of arthritis with respect to knee edema, thermal hyperalgesia, static weight bearing, leukocyte recruitment, toxicity, oxidative stress, histopathological changes in the knee joint, RT-qPCR, and proteoglycan levels in the patella. Part 3: HMC activity against TiO_2_ arthritis was tested on samples collected at the third day of arthritis on dorsal root ganglia (DRG) neuronal activity, leukocyte recruitment, cytokine release by the knee joint tissue, total reactive oxygen species (ROS) using a DCF fluorescent probe, and GSH using a fluorescent thiol tracker probe. In all figures, we opted to mention that the negative control used for knee inflammation was saline, since this was the vehicle of titanium dioxide. TiO_2_ was used to indicate the positive arthritis group, which also received the vehicle of HMC (saline, i.p.). The group indicated as HMC received the inflammatory stimulus, TiO_2_, plus HMC treatment.

**Figure 2:**
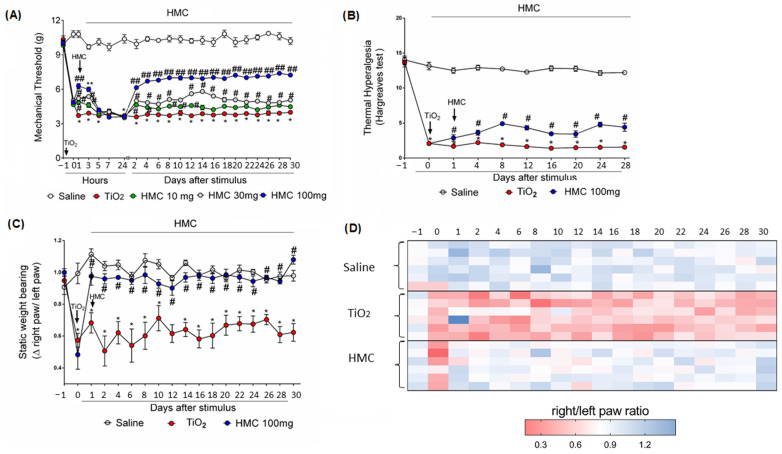
Analgesic effect of HMC on TiO_2_-induced arthritis. Mice were treated as shown in Figure 1. Mechanical threshold using an electronic pressure meter (**A**), thermal threshold of hyperalgesia using the Hargreaves apparatus (**B**), and spontaneous static paw weight distribution ratio (**C**) and its heat map (**D**) were evaluated at the indicated time points. Results are given as mean ± standard error of the mean (SEM) of 6 mice per experimental group: * *p* < 0.05 compared to the saline group, # *p* < 0.05 compared to the TiO_2_ group, ** *p* <0.05 compared to the TiO_2_ groups and doses of 10 and 300 mg/kg HMC, and ## *p* < 0.05 compared TiO_2_ groups and all other doses of HMC (Two-way ANOVA followed by Tukey’s post-test).

Figure 1 presents the schematic treatment protocol, analyses made, and time points. The mice received one intra-articular (i.a) injection of TiO_2_ (3 mg/joint), and after 24 h, the treatment with HMC started with one administration per day (10, 30, and 100 mg/kg, intraperitoneally (i.p.)) up to the 30th day (Figure 1, part 1). The TiO_2_ arthritis group presented a greater decrease in mechanical threshold compared to the saline group (Figure 2A), thus confirming that TiO_2_ induces hyperalgesia upon mechanical stimulation, as in previous studies and at the same dose [15,16]. HMC reduces the mechanical hyperalgesia triggered by TiO_2_ in a dose-dependent manner (Figure 2A). Since significantly higher activity was observed with the dose of 100 mg/kg (Figure 2A), this dose was chosen for the next experiments (Figure 1, parts 2 and 3). In the Hargreaves test of thermal hyperalgesia, HMC, again, reduced the nociceptive effect of TiO_2_ arthritis (Figure 2B). Similarly, HMC reduced the imbalance of hind paw weight distribution (right/left paw ratio values—Figure 2C, and heat map—Figure 2D), which is a measurement of non-evoked/spontaneous nociceptive behavior [33]. These results demonstrate—using varied methods—that HMC can reduce both evoked and non-evoked nociceptive behaviors triggered by articular inflammation initiated by the TiO_2_.

### 2.2. HMC Does Not Induce Kidney, Liver, or Stomach Damage

**Figure 3:**
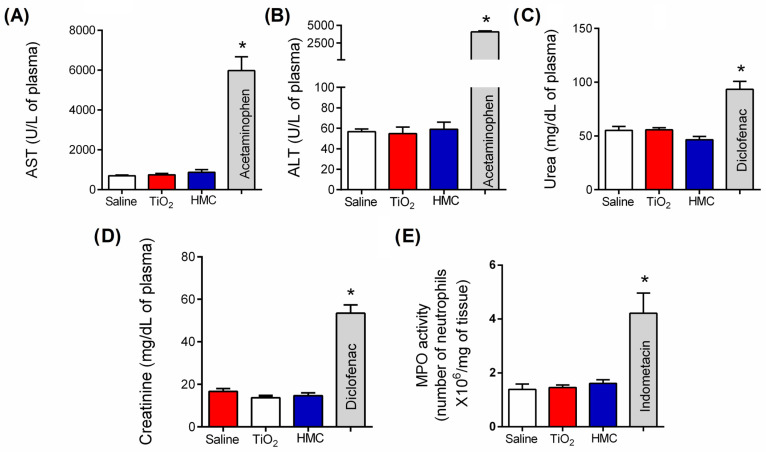
HMC does not induce liver, kidney, or stomach damage. Mice were treated as described in Figure 1. There were also treatments with drugs that induce specific organ damage, which included acetaminophen (650 mg/kg, administered orally, diluted in sterile saline, and administered once) for liver injury, diclofenac (200 mg/kg, administered orally, diluted in sterile saline, and administered once) for kidney injury, and indomethacin (2.5 mg/kg, administered i.p., diluted in tris/HCl buffer, and applied for 7 days) for stomach injury. Levels of AST (**A**), ALT (**B**), urea (**C**), and creatinine (**D**) were quantitated in the serum, and MPO activity was quantitated in samples of stomach (**E**). The results were presented as a mean ± standard error of the mean (SEM) of 6 mice per experimental group. * *p* < 0.05 compared to the saline group (one-way ANOVA followed by Tukey’s post-test).

Mice were treated daily for 30 days, as shown in Figure 1. Plasma and stomach samples were collected to determine the plasma concentration of aspartate aminotransferase (AST), alanine aminotransferase (ALT), urea, and creatinine, and the myeloperoxidase (MPO) activity in the stomach samples (Figure 3) as previously described [34,35,36]. Acetaminophen-treated mice samples were used as positive controls for liver injury (650 mg/kg, administered orally, diluted in sterile saline, and administered once). Diclofenac-treated mice (200 mg/kg, administered orally, diluted in sterile saline, and administered once) samples were used as positive controls for kidney injury. Indomethacin-treated mice (2.5 mg/kg, administered i.p., diluted in tris/HCl buffer, and applied for 7 days) were used as positive controls for stomach injury. The treatment with a dosage of 100 mg/kg of HMC did not alter the plasma concentration of AST (Figure 3A) and ALT (Figure 3B), urea (Figure 3C), and creatinine (Figure 3D), nor did it alter gastric MPO activity (Figure 3E). Thus, HMC treatment for 30 days did not induce gastric lesions or liver and kidney damage. On the other hand, acetaminophen, diclofenac, and indomethacin induced such organ lesions, respectively.

### 2.3. HMC Reduces Articular Edema and Recruitment of Leukocytes in TiO_2_ Arthritis

**Figure 4:**
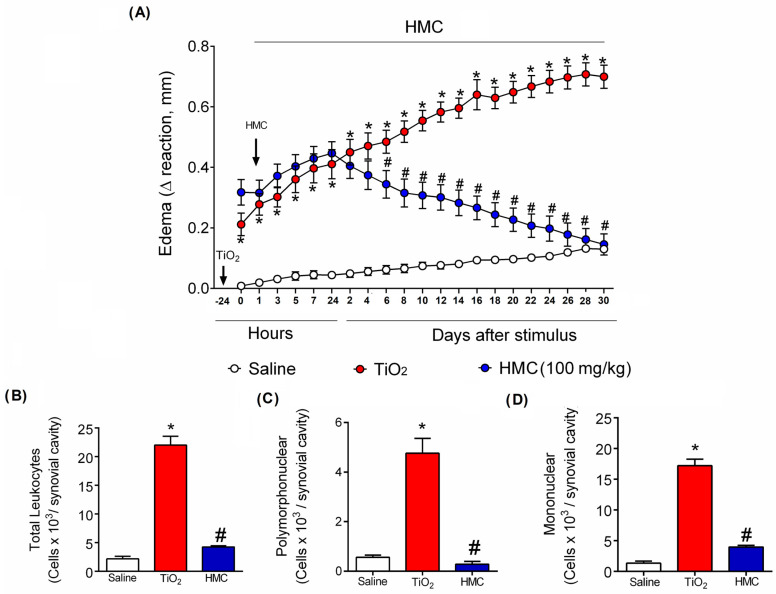
HMC reduces edema and recruitment of leukocytes caused by TiO_2_ arthritis. Mice were treated as described in Figure 1. Knee edema was evaluated 1, 3, 5, and 7 h after HMC treatment, and every other day until the end of the 30th day (**A**). Thirty days after TiO_2_ injection, the knee-joint fluid was harvested to count total leukocytes (**B**), polymorphonuclear (**C**), and mononuclear (**D**) cells. The results were presented as a mean ± standard error of the mean (SEM) of 6 mice per experimental group and were performed 2 times separately. * *p* < 0.05 compared to the saline group; # *p* < 0.05 compared to the TiO2 group (Two-way ANOVA followed by Tukey’s post-test).

Mice were treated daily for 30 days as shown in Figure 1. HMC significantly reduced the development of knee edema, with significant activity starting at the 6th day post-TiO_2_ administration (Figure 4A). Regarding leukocyte migration, it was possible to observe that HMC reduced the recruitment of total leukocytes (Figure 4B) and polymorphonuclear (Figure 4C) and mononuclear (Figure 4D) cells.

### 2.4. HMC Reduces the Histopathological Changes and Cartilage Degradation Caused by TiO_2_-Induced Arthritis

**Figure 5:**
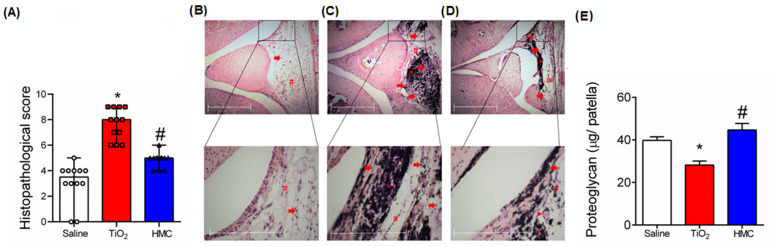
HMC reduces the histopathological changes and cartilage degradation caused by TiO_2_ arthritis. Mice were treated as described in Figure 1. Quantitative score of the histopathological changes caused by TiO_2_ in the knee joint (**A**). Representative images of knee joint sections of negative control (**B**), positive arthritis control (**C**), and HMC-treated arthritis (**D**). Arrows indicate leukocyte recruitment, the arrowheads denote neovascularization, asterisks represent TiO_2_, and squares represent synovial hyperplasia. TiO_2_ accumulation can the observed as a black pigment. DMMB assay was used to determine proteoglycan levels in the patella (**E**). Results were expressed as median + range; *n* = 12 for histopathological score. * *p* < 0.05 vs. saline; # *p* < 0.05 compared to the TiO_2_ group (Kruskal-Wallis followed by Dunn’s test) (**A**–**D**). Results are expressed as mean ± SEM; *n* = 6 animals per group per experiment for proteoglycan quantitation on patella samples. * *p* < 0.05 vs. saline; # *p* < 0.05 compared to the TiO_2_ group (one-way ANOVA followed by Tukey’s post hoc test) (**E**).

Figure 5A presents the quantitative score of the histopathological changes caused by TiO_2_ in the knee joint as well as the effect of the HMC treatment protocol as summarized in Figure 1. HMC reduced the overall histopathological score of inflammation caused by TiO_2_ (Figure 5A). The histopathological parameters analyzed are presented in Figure 5B–D, which show representative images of the knee joint sections of each experimental group. In the figures, arrows indicate leukocyte recruitment, the arrowheads denote neovascularization, asterisks represent TiO_2_, and, finally, squares indicate synovial hyperplasia. These histopathological alterations are similar to previous observations made using this model [15,16]. It is possible to observe an accumulation of TiO_2_ in the tissue, characterized by a black pigment. These histopathological changes resulted in proteoglycan degradation in the knee joint patella, which was reduced by the HMC treatment (Figure 5E).

### 2.5. HMC Reduces Oxidative Stress in TiO_2_-Induced Arthritis

**Figure 6:**
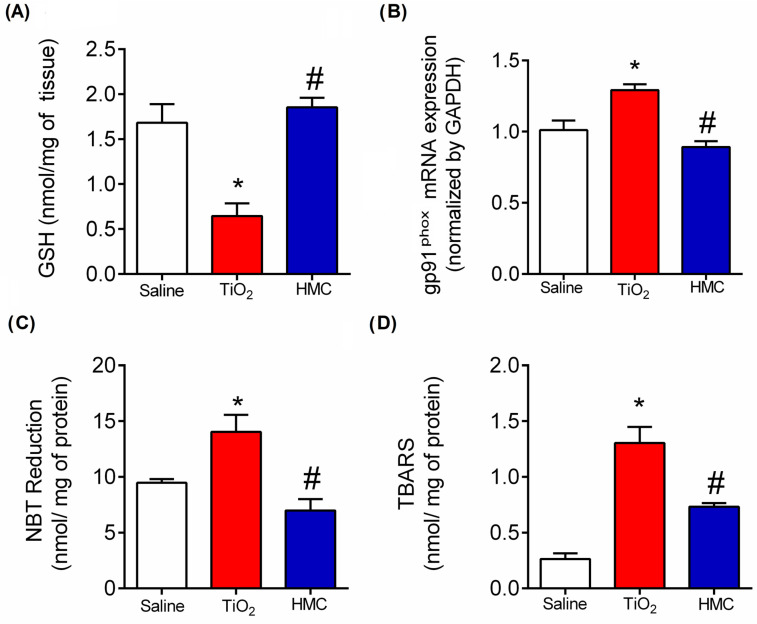
HMC reduces oxidative stress in TiO_2_-induced arthritis. Mice were treated as described in Figure 1. On the 30th day, the joint samples were harvested to quantitate GSH levels (**A**), gp91^phox^ mRNA expression (**B**), superoxide anion production (NBT reduction) (**C**), and lipid peroxidation end products (TBARS) (**D**). The results were presented as a mean ± SEM of 6 mice per experimental group. * *p* < 0.05 compared to the saline group; # *p* < 0.05 compared to the TiO2 group (one-way ANOVA followed by Tukey’s post-test).

The mice were treated daily for 30 days, as shown in Figure 1. Knee joint samples were harvested 30 days after stimulus for determination of GSH (Figure 6A), gp91^phox^ mRNA expression (Figure 6B), superoxide anion production (NBT reduction) (Figure 6C), and lipid peroxidation (TBARS) (Figure 6D). TiO_2_ decreased the levels of GSH, which is an endogenous antioxidant [34], and HMC inhibited this effect (Figure 6A). TiO_2_ increased the mRNA expression of gp91^phox^ (Figure 6B), which is an NADPH oxidase subunit involved in superoxide anion production [37]. This mRNA result was accompanied by an increase in superoxide anion production (Figure 6C) and lipid peroxidation with the formation of end products of oxidative stress (Figure 6D). Thus, these three results regarding gp91^phox^ mRNA expression (Figure 6B), superoxide anion production (Figure 6C), and lipid peroxidation (Figure 6D) are aligned and support each other. HMC reduced all these oxidative alterations caused by TiO_2_ (Figure 6), thus demonstrating its antioxidant effect, which was not solely restricted to the direct chemical antioxidant properties of its structure [29] but also involves the downmodulation of mRNA expression molecules involved in the production of free radicals such as gp91^phox^ [29,30,32].

### 2.6. HMC Reduces Acute Inflammation in TiO_2_-Induced Arthritis with Respect to Leukocyte Recruitment, Cytokine Production, and Oxidative Stress

**Figure 7:**
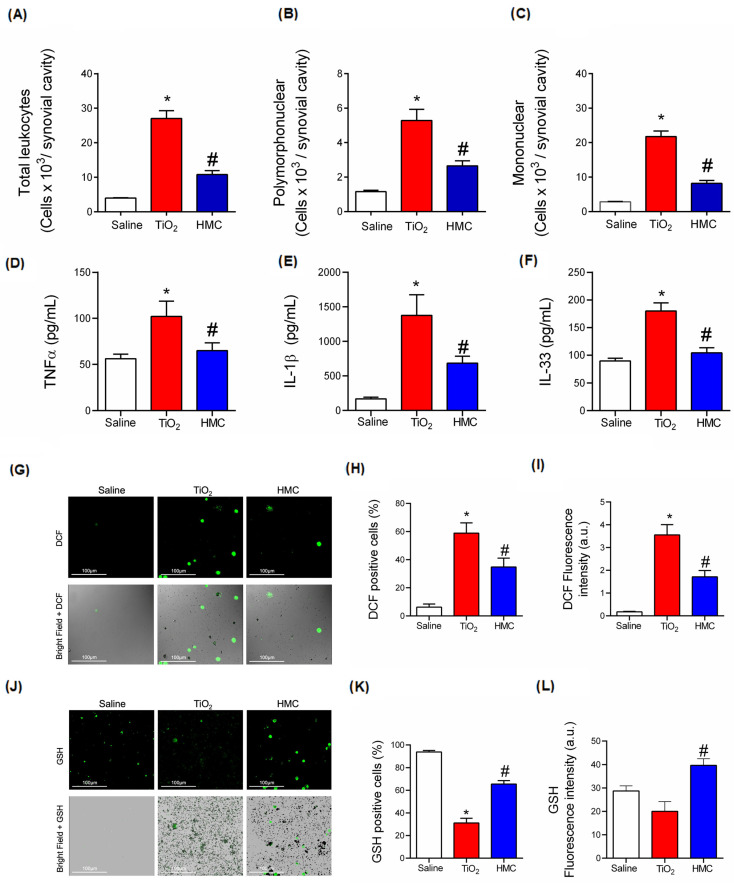
HMC reduces acute inflammation in TiO_2_-induced arthritis: leukocyte recruitment, cytokine production, and oxidative stress. Mice were treated as described in Figure 1, and samples were collected at the 3rd day after TiO_2_ injection. Samples of liquid from the synovial cavity of the knee were collected to count total leukocytes (**A**), polymorphonuclear cells (**B**), and mononuclear cells (**C**) (*n* = 6). Knee joint explants were collected after in vivo protocol depicted in Figure 1, followed by culture in media and quantitation of TNFα (**D**), IL-1β (**E**), and IL-33 (**F**) by ELISA in the conditioned media (*n* = 6). Representative images of DCF-2DA fluorescent probe assay in recruited leukocytes (**G**), total ROS-positive cells (**H**), and fluorescence intensity (**I**) (*n* = 6). Representative images of GSH quantitation in recruited leukocytes using a thiol-sensitive fluorescent probe (**J**), total GSH-positive cells (**K**), and fluorescence intensity (**L**) (*n* = 6). The results were presented as a mean ± SEM of 6 mice per experimental group (**A**–**F**) or 6 pools of 4 mice per pool per experimental group (**G**–**L**). * *p* < 0.05 compared to the saline group; # *p* < 0.05 compared to the TiO_2_ group (one-way ANOVA followed by Tukey’s post-test).

The results of the previous Figure 2, Figure 4 and Figure 5 demonstrate that HMC reduces TiO_2_-induced arthritis pathology, inflammatory leukocyte recruitment, and pain. These effects involve a reduction in oxidative stress (Figure 6), which is aligned with the antioxidant activity of HMC [29]. Figure 2 and Figure 4 also demonstrate that symptoms and inflammatory events such as hyperalgesia and edema can be observed early in the course of TiO_2_-induced arthritis. To reduce the time that the mice suffered from inflammation, as the chronic treatment protocol with HMC had already been demonstrated and showed no signs of common side effects (Figure 3), we evaluated the effect of HMC 3 days after TiO_2_ administration, as depicted in Figure 1, part 1. HMC could also reduce the recruitment of total leukocytes (Figure 7A) and polymorphonuclear (Figure 7B) and mononuclear (Figure 7C) cells caused by TiO_2_-induced arthritis. At this time point, HMC also inhibited the production of the pro-inflammatory and hyperalgesic cytokines TNFα (Figure 7D), IL-1β (Figure 7E), and IL-33 (Figure 7F), thus further confirming that inflammation was ongoing at this time point and that HMC can inhibit it. Using fluorescent probes, we also observed that TiO_2_ increases total ROS production, as shown in the representative images of the recruited leukocytes (Figure 7G); the total number of ROS-positive cells (Figure 7H); and the intensity of fluorescence (Figure 7I). Using another fluorescent probe, we also observed—as shown in the representative images (Figure 7J)—that TiO_2_ reduces the number of leukocytes that react positively towards the total thiol groups, which are mostly composed of GSH, and that HMC maintained the number of GSH-positive leukocytes in the knee joints (Figure 7K). When considering the total fluorescence intensity, TiO_2_ did not cause a significant reduction in GSH levels, but this is likely because the TiO_2_ group has approximately seven-fold more recruited leukocytes in the knee joints than the saline group (Figure 7L). Thus, when analyzing the total fluorescence (Figure 7L), the difference between the saline and TiO_2_ groups is less evident in terms of total fluorescence intensity. However, even with the difference in the recruitment of leukocytes, HMC could significantly increase the GSH levels compared to the TiO_2_-induced arthritis control (Figure 7L). Thus, despite having a lower count of recruited leukocytes than the TiO_2_ group, the HMC group presented more GSH than the TiO_2_ group (Figure 7J–L). These results, shown in Figure 7, suggest the feasibility of detecting the oxidative balance in the recruited leukocytes, which aligns with the results of Figure 6 that approached the oxidative balance in the knee joint tissue.

### 2.7. HMC Reduces Nociceptor Sensory Neuron Activation Caused by TiO_2_-Induced Arthritis

**Figure 8:**
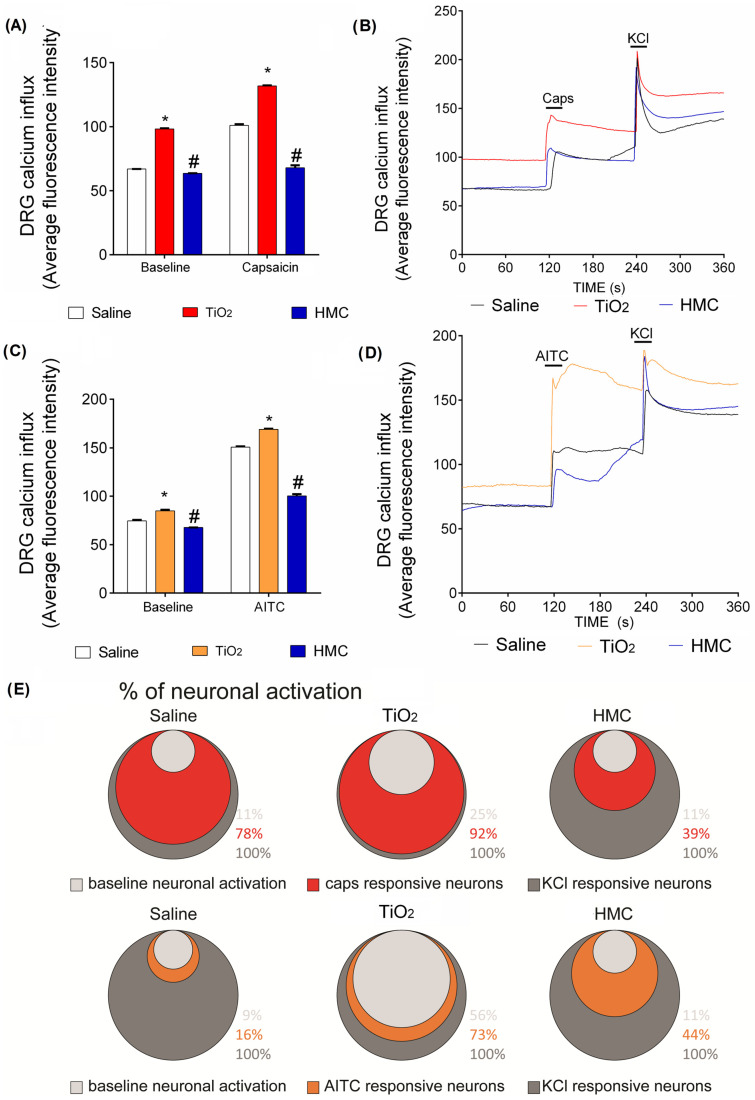
HMC reduces the activation of TRVP1^+^ and TRPA1^+^ nociceptor sensory neuron in TiO_2_-induced arthritis. Mice were treated as described in Figure 1. DRGs were collected to isolate primary afferent sensory neurons at the 3rd day after TiO_2_ injection. Calcium levels were determined in basal conditions and after capsaicin-induced stimulation of TRPV1+ neurons, as shown in the representative traces (**A**) and mean ± SEM (**B**). Calcium levels were also determined in basal conditions and after AITC stimulation of TRPA1^+^ neurons, as shown in the representative traces (**C**) and mean ± SEM (**D**). KCl was used as a control of depolarization and viability (**A**–**E**). Venn diagram presents the percentage of responsive neurons in basal condition, upon stimulation with capsaicin or AITC, and KCl-responsive neurons (**E**). In addition to these three steps, there are three groups (saline plus vehicle—indicated as Saline; TiO_2_ plus vehicle—indicated as TiO_2_; and TiO_2_ plus HMC—indicated as HMC) (H). The results were presented as a mean ± SEM of 6 pools of DRGs per experimental group, and each pool derived from 6 mice. * *p* < 0.05 compared to the saline group; # *p* < 0.05 compared to the TiO_2_ group (two-way ANOVA followed by Tukey’s post-test).

Figure 7 confirms that inflammation is fully active at the 3rd day post-TiO_2_ administration. A final point that we endeavored to understand was whether HMC can inhibit neuronal function, thereby explaining its analgesic mechanism. To this end, dorsal root ganglia (DRG) neurons corresponding to the L4–L6 vertebrae of the TiO_2_-induced arthritis mice were harvested, since these DRGs are the cellular bodies of the sensory neurons innervating the knee joint. These DRG neurons were isolated, cultured, and loaded with a fluorescent probe to detect intracellular calcium in the live cells. TiO_2_-induced arthritis caused an enhanced basal level of calcium in the DRG neurons compared to the saline non-inflamed group (Figure 8). It is interesting that TiO_2_-induced arthritis also enhanced the response to capsaicin, a TRPV1 agonist (Figure 8A,B,E); thus, TiO_2_-induced arthritis enhances the activation of TRPV1^+^ nociceptive neurons. TiO_2_-induced arthritis also enhanced the activation of TRPA1^+^ neurons (Figure 8C–E). HMC treatment reduced the basal neuronal activation caused by TiO_2_-induced arthritis as well as the responsiveness of TRPV1^+^ (Figure 8A,B,E) and TRPA1^+^ (Figure 8C–E) DRG neurons, which are two important subsets of nociceptive neurons. To our knowledge, this is the first evidence that HMC reduces the activation of TRPV1^+^ and TRPA1^+^ neuronal populations (Figure 8).

## 3. Discussion

Arthroplasty is a surgical procedure conducted to totally or partially replace a dysfunctional joint. This procedure is important in order to reestablish patients’ quality of life, mitigate pain, and recover joint function and mobility. Despite arthroplasty’s importance, this procedure results in failure in 15% of cases, increasing to 40% depending on the study [8,9]. The wearing process causes the release of TiO_2_ from the prosthesis, and the inflammatory response caused by TiO_2_ contributes to the aseptic loosening of the prosthesis, pain, and, ultimately, leads to the need for revision arthroplasty [1,2]. In the present study, we investigated the effect and mechanisms of action of HMC in TiO_2_-induced arthritis while considering what has been previously demonstrated in terms of the activity and mechanisms of action of this flavonoid [27,28,29,30,31,32]. We observed that the HMC treatment was effective in reducing the disease parameters caused by TiO_2_, which included hyperalgesia upon mechanical and thermal stimulation; the un-evoked, unbalanced weight distribution between the hind paws; knee edema; neutrophil and mononuclear leukocyte recruitment; histopathological inflammatory changes; and proteoglycan degradation. The beneficial effects of HMC against these pathological changes were based on reductions in oxidative stress, cytokine production, and nociceptor sensory neuron activation.

HMC is already in clinical use under the commercial name Cyclo 3^®^ Fort. This remedy is a mixture of *Ruscus aculeatus* extract, vitamin C, and HMC that is applied in the treatment of chronic venous diseases [38,39]. Clinical trials that have evaluated Cyclo 3^®^ Fort support its therapeutic activity in chronic venous and lymphatic insufficiency-related symptoms and edema [40]. HMC is an interesting flavonoid since its structure is a mixture of a flavanone and a chalcone. HMC is a semi-synthetic flavonoid-based on hesperidin (hesperidin-7-rhamnoglucoside), which is transformed into HMC by undergoing methylation in alkaline media. This partial synthesis generates a molecule with higher water solubility [41]. In addition to its uses as a component of Cyclo 3^®^ Fort, pre-clinical data support that HMC is an analgesic and anti-inflammatory flavonoid [38,39]. The evidence in mouse models demonstrates that HMC treatment reduces inflammation and nociceptive-like behavior triggered by varied stimuli (e.g., carrageenan, monosodium urate crystals, zymosan, LPS, UVB skin inflammation, Complete Freund’s Adjuvant, and colitis) [27,28,29,30,31,32]. Under these experimental conditions, HMC reduces the recruitment of leukocytes, thus contributing to reductions in inflammation and pain [42]. In the present study, HMC also reduced leukocyte recruitment with respect to neutrophils and mononuclear leukocytes.

Another consistent finding regarding HMC activity is the compound’s antioxidant activity, which is intrinsic to its chemical dual flavonoid structure [43,44]. HMC also reduced the oxidative stress caused by TiO_2_-induced arthritis, as shown by increased superoxide anion production and oxidative stress end products such as TBARS [31]. Using an opposite approach, we observed that HMC also maintained the levels of endogenous antioxidants such as GSH. The antioxidant outcome of HMC treatment is not solely related to its chemical antioxidant groups, such as hydroxyl groups [29], but it is also related to the regulation of gene expression [28,29]. HMC reduced the mRNA expression of gp91^phox^ in the TiO_2_-induced arthritis models; notably, gp91^phox^ is a subunit of the enzyme NADPH oxidase that produces the superoxide anion [29,32]. The effect of HMC against gp91^phox^ mRNA expression aligned well with its activity of reducing superoxide anion production and lipid peroxidation in the TiO_2_-induced arthritis models. Reactive oxygen species (ROS) induce pain, edema, and leukocyte infiltration in tissues [43,44,45]. ROS induce histopathological changes similar to TiO_2_-induced arthritis [15,16,32,46,47]. Leukocytes produce ROS that contribute to tissue damage, which includes the proteoglycan degradation of cartilage [15,47]. We also observed that HMC reduced the total ROS production by the recruited leukocytes in the knee joints. Observing the opposite side, HMC increased GSH levels and positive cells, indicating that HMC increases cellular antioxidant defenses in the leukocytes present in the synovial cavity. Reducing the oxidative stress in recruited leukocytes diminishes their capability of inducing tissue damage [47]. Aligning with HMC’s biological activity, its therapeutic effect on TiO_2_-induced arthritis included reducing pain, edema, leukocyte recruitment, and proteoglycan degradation.

HMC is a multi-target drug. HMC can reduce the activation of the pro-inflammatory transcription factor NFkB [27,28,29,30,31,32]. HMC reduces the phosphorylation of NFkB in varied disease models in vivo [31,32] and in vitro in RAW 264.7 macrophage cultures [28]. In silico and proof-of-concept biological results demonstrate that HMC binds to the Ser276 of NFkB and inhibits its activation/phosphorylation [28]. In line with this evidence, HMC reduced the production of TNFα, IL-1β, and IL-33 in the TiO_2_-inflamed knee joint. These cytokines have been shown to induce leukocyte recruitment [48,49], oxidative stress [50,51], edema [52], and pain [53,54]. Thus, the inhibition of cytokine production by HMC seems to be a contributory activity to its mechanism of action. HMC can also induce Nrf2 mRNA expression and that of its down-stream targets HO-1 and NQO1. Nrf2 is a transcription factor involved in inducing antioxidant and detoxifying responses as well as reducing inflammation [55]. Furthermore, HMC reduces the protein levels of Keap-1, which is responsible for limiting Nrf2’s cytoplasmic levels by inducing its ubiquitination and proteasomal degradation [55]. These mechanisms of HMC might explain its beneficial activity in terms of TiO_2_-induced arthritis. Importantly, the 30-day treatment with HMC did not induce hepatic, renal, or gastric lesions, which are common side effects of non-steroidal anti-inflammatory drugs [34,35,36,55]. In fact, the opposite occurs, since HMC can reduce the renal damage caused by the non-steroidal anti-inflammatory drug diclofenac by inducing Nrf2 activation [55].

Capsaicin has been shown to induce the production of reactive oxygen species [56] and HMC reduces the nociceptive behavior induced by capsaicin administration in vivo [27]. Capsaicin is an agonist of TRPV1 ion channels, which are expressed by most nociceptive neurons known as C fibers. AITC is an agonist of the TRPA1 receptor, which is mostly co-expressed by TRPV1^+^ C fibers and, in a small proportion, by other neuronal populations. Thus, TRPV1^+^ and TRPA1^+^ DRG neurons represent important subsets of nociceptive neurons [57,58,59,60,61]. In the present study, we demonstrate, for the first time, that TRPV1^+^ and TRPA1^+^ DRG nociceptive neurons are activated in TiO_2_-induced arthritis. Furthermore, for the first time, we demonstrate that HMC reduces the neuronal activation of TRPV1^+^ and TRPA1^+^ nociceptive neurons. Therefore, we have provided novel physiopathological evidence regarding TiO_2_-induced arthritis and the pharmacological mechanisms of HMC.

Unfortunately, there is limited evidence regarding the pharmacokinetics of HMC. One study investigated the pharmacokinetics of ^14^C-labeled HMC [62]. An empiric dose of 10 mg/kg of ^14^C-HMC was administrated orally. The ^14^C-HMC reached its blood level peak within 1–2 h after administration, but similar levels were maintained up to 8 h. Excretion occurred mainly through feces, but also via urine within 24 h of administration. When ^14^C-HMC was administrated in the Cyclo 3^®^ formulation, higher blood levels were achieved compared to the administration of solely ^14^C-HMC, and the blood levels of ^14^C-HMC remained high up to 24 h instead of up to 8 h [62]. This study [62] presents a different experimental condition compared to ours since we used a 100 mg/kg administration via the intraperitoneal route, which achieves higher absorption than the oral route. However, the cited study provides data stating that HMC is absorbed and that this absorption can be improved [62]. To our knowledge, there is no study investigating whether HMC reaches the central nervous system. However, there is evidence that the intravenous administration of HMC reduces the development of granulomas and the production of inflammatory molecules in the spinal cord in a model wherein *Mycobacterium tuberculosis* was administrated into the spinal cord [63]. This evidence supports the notion that the peripheral administration of HMC can promote this compound’s activity in the central nervous system. Under our experimental conditions, HMC reduced the activity of DRG neurons, which does not necessarily mean that HMC reaches the central nervous system. The primary afferent sensory neurons whose cellular bodies are in the DRG project their axons to the spinal cord and peripheral tissues. Therefore, these primary afferent sensory neurons represented by the DRG neuronal culture have access, through their axons, to cellular and biochemical processes transpiring in the peripheral tissues as well as events occurring in the central nervous system. There is even evidence of retrograde as well as anterograde axonal transport of cytokines such as TNFα through the primary afferent sensory neurons, and that peripherally produced TNFα and its receptors will reach the DRG [64,65]. The primary afferent nociceptor sensory neurons have also been described to present a pharmacodynamic property termed teleantagonism. An example of this phenomenon is that the sensitization of primary afferent nociceptor sensory neurons can be induced by the injection of a nociceptive stimulus by the intrathecal route, and this nociceptor sensitization can be inhibited by intraplantar (subcutaneous injection in the plantar face of the paw) treatment with a receptor antagonist or an enzyme inhibitor [66]. Thus, HMC does not need to reach the central nervous system to inhibit ion channels expressed by primary afferent sensory neurons; rather, it acts on their axons. Accordingly, the lack of direct evidence of HMC’s presence in the central nervous system does not affect the present results or the proposed neuronal mechanism.

## 4. Materials and Methods

### 4.1. General Experimental Procedures

In the first series of experiments, mice (*n* = 6 per group per experiment) were submitted to a dose–response experiment of hesperidin methyl chalcone (HMC) treatment. Mice were stimulated with an intra-articular (i.a.) injection with 3 mg of TiO_2_ (suspended in 10 µL of sterile saline solution; 0.9% NaCl) per knee joint. Mechanical hyperalgesia and edema were evaluated 24 h after i.a. administration of TiO_2_ to assess the arthritis model’s induction. Subsequently, after 24 h, mice were treated with HMC (10, 30, 100, or 300 mg/kg; administered intraperitoneally (i.p.); and diluted in sterile saline solution of 0.9% NaCl) and mechanical hyperalgesia and edema were evaluated 1, 3, 5, 7, and 24 h after HMC treatment in the first day. Then, mechanical hyperalgesia was evaluated every other day 1 h after HMC treatment until the 30th day. Immediately after the measurements on the 30th day, mice were anesthetized and euthanized, and the synovial fluid of knee joint cavities was harvested for an evaluation of leukocyte migration. A 100 mg/kg dose of HMC was chosen based on the results regarding mechanical hyperalgesia and was used in the following experiments. Thermal hyperalgesia (Hargreaves test), static weight imbalance between the hind paws, and knee edema were evaluated for 30 days. Blood and stomach samples were harvested to evaluate plasmatic levels of AST, ALT, urea, and creatinine, as well as the stomach for myeloperoxidase (MPO) activity. The knee joints were harvested to evaluate oxidative stress (gp91^phox^ mRNA expression (RT-qPCR), superoxide anion (nitroblue tetrazolium reduction levels), reduced glutathione (GSH), and lipid peroxidation (TBARS)). On the 30th day, patella samples were harvested to quantitate proteoglycan levels by a colorimetric assay. On the 3rd day, samples of synovial fluid of knee joint cavities were collected to evaluate leukocyte recruitment as well as total ROS production and GSH levels in those leukocytes; knee joint explants were collected and cultured for 2.5 h to quantitate cytokines in the conditioned media; and DRG were collected calcium imaging to assess neuronal activation.

### 4.2. Animals

Male, Swiss mice weighing between 20–25 g from the Londrina State University, Londrina, Paraná, Brazil, were used in this study. Mice were housed in standard clear plastic cages with water and food provided ad libitum and in a light/dark cycle of 12/12 h and a controlled temperature (21 °C). Mice were maintained in the vivarium of the Department of Pathology of Londrina State University for at least two days before experiments. Mice were used only once and were acclimatized to the testing room at least 1 h before the experiments, which were conducted during the light cycle. Animal care and handling procedures were in accordance with the International Association for Study of Pain (IASP) guidelines and approved by the Londrina State University Ethics Committee on Animal Research and Welfare (process number 20466.2015.58). All efforts were made to minimize the number of animals used and their suffering.

### 4.3. Test Compounds

The compounds used in this study were saline solution (NaCl 0.9%; Frenesius Kabi Brasil Ltd.a, Aquiraz, Brazil), Ethylenediaminetetraacetic acid disodium salt (EDTA; Synth, Diadema, Brazil), and hesperidin methyl chalcone (HMC, 98% purity, Santa Cruz Biotechnology, Santa Cruz, CA, USA). Titanium dioxide was purchased from Synth (Diadema, Brazil) and particle size was <1 µm with an average of 862.2 nm as determined by size distribution analysis (Malvern Instruments Ltd., Malvern, UK). Immediately before the injections, TiO_2_ was suspended in sterile saline (10 μL). HMC (10, 30 or 100 mg/kg, diluted in saline) and saline was administered i.p. at a volume of 200 µL.

### 4.4. Evaluation of Articular Mechanical Hyperalgesia

The articular mechanical hyperalgesia of the femorotibial joint was evaluated as previously described [33]. Briefly, in a quiet room, mice were placed individually in acrylic cages (12 × 10 × 17 cm) with a wire grid floor 15–30 min before the test for environmental adaption. Force was applied only when animals were quiet, did not display exploratory movements or defecation, and were not resting on their paws. An electronic pressure-meter test consisting of a hand-held force transducer fitted with a polypropylene tip (electronic von Frey Anesthesiometer; Insight, Ribeirão Preto, Brazil) was used to evaluate mechanical articular nociception. For this model, a large tip (4.15 mm^2^) was adapted to the probe. An increasing perpendicular force was applied to the central area of the plantar surface of the hind paw to induce a flexion movement of the femorotibial joint followed by paw withdrawal. A tilted mirror below the grid provided a clear view of the hind paw. The electronic pressure-meter apparatus automatically recorded the intensity of the force applied when the paw was withdrawn. The test was repeated until the subsequent consistent measurements (i.e., the variation among these measurements was less than 1 g) were obtained. The flexion-elicited mechanical threshold was expressed in grams (g) [33].

### 4.5. Thermal Hyperalgesia

Thermal hyperalgesia was evaluated with the aid of the Hargreaves algesimeter (Ugo Basile, Gemonio, Italy). Briefly, in a quiet room, the animals were placed individually in acrylic cages (12 × 10 × 17 cm) with a glass floor 15 to 30 min before the environmental adaptation test. Heat was only applied when the animals had all four legs on the base of glass, did not exhibit exploratory movements or defecation, and were not resting on their paws. The standardized paw for the measurement was the right paw due to its proximity to the induction area with TiO_2_. An infrared thermal force of 30 nm was applied to the central region of the animal’s paw, and the time that the animal remained in the same position at which the heat was generated was analyzed. The moment the animal made an initial movement, the device finished the measurement in seconds. Heat should only be applied for a maximum of 20 s; accordingly, this was applied as the cut-off point.

### 4.6. Static Weight Bearing 

The animals were individually conditioned in an acrylic device positioned forward, with each mouse’s front paws supported at the front, while their hind legs were supported by a weight measurement sensor (g). The test began at the moment the animal was immobilized; then, weight measurement was performed for 10 s. At the end of this period, the weight of the left and right paw was measured, with the right being relative to the stimulus. This test evaluates the weight distribution between a mouse’s hind legs. While an unstimulated animal distributes its weight equally between the two legs, the ratio of weight distribution between the stimulated and unstimulated paw is a measure of the level of discomfort in the stimulated paw. Via the continuous measurement of the weight supported by each paw, the Static Weight Bearing (Bioseb, Vitrolles, France) method allows for an objective measurement of spontaneous pain by assessing postural balance [67].

### 4.7. Stomach Toxicity Assay (Myeloperoxidase) 

The Myeloperoxidase (MPO) activity was used to determine stomach toxicity via a colorimetric assay, as this is a reliable marker for non-steroidal anti-inflammatory-drug-induced stomach lesions [36,67]. Samples of the stomach were collected in 50 mM K_2_HPO_4_ buffer (pH 6.0) containing 0.5% hexadecyl trimethylammonium bromide (HTAB) and kept at −86 °C until use. Frozen samples were homogenized using a tissue turrax (Tissue-Tearor 985370, BioSpec Products, Bartlesville, OK, USA), centrifuged (2 min, 16,000× *g*, 4 °C), and the resulting supernatant was assayed using a spectrophotometer (Multiskan GO Microplate Spectrophotometer, Thermo Scientific, Vantaa, Finland) to determine MPO activity at 450 nm. The MPO activity of samples was compared to a standard curve of neutrophils. Briefly, 15 µL of sample was mixed with 200 µL of 50 mM phosphate buffer (pH 6.0) containing 0.167 mg/mL O-dianisidinedihydrochloride and 0.0005% hydrogen peroxide. The results were presented as MPO activity (number of neutrophils × 106/mg of tissue).

### 4.8. Liver and Kidney Toxicity Assays 

Blood was harvested in microtubes containing 50 µL of the anticoagulant EDTA (5000 IU/mL), centrifuged (200× *g*, 10 min, 4 °C), and the plasma was separated. In order to determine enzymatic activities of AST and ALT as indicators of hepatotoxicity compared to acetaminophen, and to ascertain urea and creatinine levels as indicators of nephrotoxicity compared to diclofenac, plasma samples were processed according to the manufacturer’s instructions (Labtest Diagnóstico S.A., Lagoa Santa, Brazil). Results were presented as U/mL (AST and ALT) or mg/dL (urea and creatinine) of plasma [34,35].

### 4.9. Articular Edema Measurements 

Articular edema of the femorotibial joint was assessed by measuring the joint’s transverse diameter using calipers (Digmatic Caliper, Mitutoyo Corporation, Kanagawa, Japan). Thickness values of the femorotibial joint were expressed as the difference between the diameters measured before (basal value) and after intra-articular injection of TiO_2_ in millimeters (mm) [33].

### 4.10. Leukocyte Migration Evaluation 

The total and differential counts of recruited leukocytes to the intra-articular space were determined as previously described [15,16]. Briefly, knee joint cavities were washed with saline containing Ethylenediaminetetraacetic acid (EDTA), which was recovered to evaluate total and differential cell counts. Total cell counts were performed in Neubauer chamber using Turk solution, and differential cell counts (100 cells per slide) were performed in slices stained with a panoptic kit (Laborclin, Pinhais, Brazil) under a light microscope (Olympus CX31RTSF, Tokyo, Japan). Results were expressed as total leukocytes, polymorphonuclear, and mononuclear cells (cells × 10^3^/knee joint).

### 4.11. Histological Processing 

The samples of the femorotibial joint were collected, fixed in buffered formalin, and submitted to histological processing for dehydration with graduated alcohol baths (Dinâmica Química Contemporânea, São Paulo, Brazil), diaphanization with xylol baths (Synth, Diadema, Brazil), and impregnation in paraffin (Synth, Diadema, Brazil). Sections of 7 μm were made using a microtome. The slides with the sections were submitted to hematoxylin (Laborclin, Pinhais, Brazil) and eosin (Laborclin, Pinhais, Brazil) staining. Through Hematoxylin and Eosin (HE) staining, it was possible to observe the tissue and perform an analysis according to the clinical score, wherein synovial hyperplasia, neovascularization, and inflammatory infiltrate were evaluated based on a score from 0 to 3, in which the severity of tissue degradation corresponds to the increasing levels, with 0 for no alteration and 3 for the most severe alteration, in which case it may be a significant increase in hyperplastic tissue, new vessels, and inflammatory infiltrate that damages the tissue. Six semi-serial sections of each animal were analyzed for each group. After the analysis, the average of each animal was taken [15,16].

### 4.12. Proteoglycan Assay 

Proteoglycan concentration was determined as previously described [49]; the patellae were carefully collected from mice and fixed with formaldehyde (4%) overnight using a shaker. Subsequently, the patellae were transferred into a solution of formic acid (5%) and incubated for 4 h using a shaker for decalcification. Then, each patella sample was placed into 100 µL of papain digestion buffer consisting of a papain suspension (5 mg/mL) in calcium and magnesium-free PBS with 5 mM of cysteine and 10 mM EDTA at pH 7.4. Samples were sealed and incubated in a humidified container at 60 °C for 16 h. After reaching room temperature, samples were centrifuged for 10 min at 1000× *g* to collect the condensation droplets. Next, 50 µL volumes of the supernatants and of serial chondroitin sulfate solutions (standard curve; 50–1000 mg/mL) were placed into 96-well plates. The chondroitin sulfate standard solution was also incubated with papain digestion buffer. Then, 300 µL of a 1,9-dimethylmethyleneblue (DMMB; 50 mg/L, Polysciences, Inc., Warrington, PA, USA) solution was added to each well, and proteoglycan content was determined by a spectrophotometer reading at 525 nm (Multiskan GO Microplate Spectrophotometer, ThermoScientific, Vantaa, Finland). The glucosaminoglycan (GAG) content was calculated from the standard curve of chondroitin 6-sulfate sodium salt from shark cartilage. The DMMB solution was prepared by dissolving 50 mg of DMMB in 5 mL of ethanol and diluting the solution to a volume of 1000 mL with 0.2% (*w*/*v*) sodium salt of formic acid (sodium formate buffer, HCOONa) at pH 3.5. The results were presented as proteoglycan in microgram/patella.

### 4.13. RT-qPCR 

Samples were homogenized in TRIzol reagent, and total RNA was extracted by using the SV Total RNA Isolation System (Promega, Madison, WI, USA). The purity of total RNA was measured using a spectrophotometer (Multiskan GO Microplate Spectrophotometer, Thermoscientific, Vantaa, Finland) and the wavelength absorption ratio (260/280) was between 1.8 and 2.0 for all preparations. Reverse transcription of total RNA to cDNA and qPCR was carried out using GoTaq^®^ 2-Step RT-qPCR System (Promega) and specific primers (gp91^phox^ and GAPDH). qPCR reaction was performed in StepOnePlusTM Real-Time PCR System (Applied Biosystems^®^, Waltham, MA, USA). The relative gene expression was measured using the comparative 2-(∆∆Cq) method. The primers used were: gp91^phox^, sense: 5′-AGCTATGAGGTGGTGATGTTAGTGG-3′, antisense: 5′-CACAATATTTGTACCAGACAGACTTGAG-3′; GAPDH sense: 5′-CATACCAGGAAATGAGCTTG-3′, antisense: 5′-ATGACATCAAGAAGGTGGTG. The expression of GAPDH mRNA was used as a reference gene, and the results were expressed as mRNA expression (normalized to GAPDH) [15,16].

### 4.14. Measurement of Reduced Glutathione (GSH) Level 

Knee-joint samples were collected and maintained at −80 °C for at least 48 h; afterwards, they were homogenized with 200 μL of 0.02 M EDTA. The homogenate was mixed with 25 μL of trichloroacetic acid 50% and was homogenized three times over 15 min. The mixture was centrifuged (15 min × 1500× *g* 4 °C). The supernatant was added to 200 μL of 0.2 M TRIS buffer, at pH 8.2, and 10 μL of 0.01 M DTNB. After 5 min, the absorbance was measured at 412 nm (Multiskan GO, Thermo Scientific, Vantaa, Finland) against a blank reagent with no supernatant. A standard GSH curve was generated. The results are expressed as GSH per mg of protein [68].

### 4.15. Nitrobluetetrazolium Reduction 

The superoxide anion production was determined by the reduction of the redox dye Nitroblue tetrazolium (NBT). Frozen knee joint tissue from mice was homogenized with 500 µL of saline using an ultra-turrax (Tissue-Tearor 985370, BioSpec Products, Bartlesville, OK, USA) and centrifuged (10 min, 3300× *g*, 4 °C); then, 50 µL of the homogenate was placed in 96-well plate, followed by the addition of 100 µL of nitro blue tetrazolium solution (1 mg/mL) (NBT, Sigma-Aldrich, St. Louis, MO, USA), and it was maintained at 37 °C in a warm bath for 5 min. The supernatant was removed, and the formazan that had precipitated was then solubilized by adding 120 µL of 2 M KOH and 120 µL of dimeltisulfoxide (DMSO). The optical density was measured using a microplate spectrophotometer reader (Multiskan GO Microplate Spectrophotometer, Thermoscientific, Vantaa, Finland) at 600 nm. The NBT reduction levels were corrected according to the total protein concentration and the results were presented as NBT reduction (OD/mg of protein) [68].

### 4.16. Lipid Peroxidation 

Tissue lipid peroxidation was assessed by the levels of thiobarbituric acid-reactive substances (TBARS) [40]. For this assay, TCA 10% was added to the homogenate and the mixture was centrifuged (3 min, 1000× *g*, 4 °C) to precipitate proteins. The protein-free supernatant was then separated and mixed with TBA (0.67%). The mixture was kept in a water bath (15 min, 100 °C). Malondialdehyde (MDA), an intermediate product of lipid peroxidation, was determined by the difference between absorbance at 535 and 572 nm using a microplate spectrophotometer reader. The TBARS were corrected according to the total protein concentration and results were presented as TBARS (µmol of MDA/mg of protein) [68].

### 4.17. Intracellular GSH Detection in Recruited Leukocytes Using a Thiol-Tracker Fluorescent Probe 

GSH, the most abundant non-protein thiol, was quantitated using an intracellular fluorescent thiol-tracker detection probe (Thiol Tracker, T10095, Invitrogen, Waltham, MA, USA). After the standardized three-day TiO_2_ induction (3 mg/joint), HMC treatments (100 mg/kg, two days of treatment), and the euthanasia of the mice, knee joint capsules were opened and recruited leukocytes harvested by washing the joints three times with 3.3 µL, which was added to 0.1 mL of FACs buffer. Total leukocyte count was performed with 10 µL prior to sample centrifugation (5 min, 0.1× *g*, and 20 °C). The supernatant was discarded, and cells were resuspended with incomplete RPMI medium and allowed to attach to the center of the 15mm glass bottom cell culture dish for 15 min before adding 3 mL of incomplete RPMI. Plates were kept for 30 min in an incubator at 37 °C with 5% CO_2_ followed by 30 min incubation with the thiol fluorescent probe (1:1000—20 mM) diluted on incomplete RPMI. Incubations and analysis were performed in sets of three culture dishes (saline, TiO_2_, and HMC groups) to assure equal experimental conditions. Cells were washed three times with HBSS Ca^2+^ Mg^2+^, which was also added for visualization and quantification by confocal microscopy (TCS SP8, Leica, Mannheim, Germany) using 63× augmentation objectives (UV light settings/405 nm excitation, 525 nm emission) and Leica X software (LAS X, Leica, Mannheim, Germany). Quantitation of positive and negative cells was performed through the random selection of 300 cells per plate per group on bright field images; selected cells that were not visualized posteriorly on fluorescent fields were considered negative, and fluorescence intensity was compared selecting the whole image field. Consequently, four pictures of each culture dish were obtained. Each culture dish contained recruited leukocytes pooled from the knee joints of 4 mice, and there were 6 pools per group. Analysis was performed by two independent observers blinded to treatments. Fluorescence-positive cells were represented in percentages, and fluorescence intensity was represented in arbitrary units of field fluorescence using the same settings for every picture throughout the experiment for all groups (saline, TiO_2_, and HMC groups) [67].

### 4.18. Intracellular Total ROS Detection by DCF Probe in Recruited Leukocytes

The fluorescent probe H2DCFDA (H2-DCF and DCF D399, Invitrogen) was used to measure the intracellular levels of total ROS. We followed the same protocol for sample collection and processing as described for the GSH probe assay. A pool of four knee joint articular exudates (10 µL per synovia obtained by washing knee joint cavities three times with 3.3 µL of FACs buffer) was harvested after three days of induction with TiO_2_ (3 mg/joint) and two treatments with 100 mg/kg of HMC (24 h apart). Total leukocytes were counted before the centrifugation step (5 min, 0.1× *g*, and 20 °C) and then, all the following steps were conducted according to GSH probe detection methodology. The H2DCFDA 20 mM probe was diluted in RPMI incomplete medium (1:200) and incubated for 30 min, washed three times, and HBSS Ca^2+^ Mg^2+^ was added for analysis with confocal microscopy (Ex/Em: ∼492–495/517–527 nm). Quantification parameters were the same as those applied in the GSH assay. Positive and negative cells’ quantitation was performed through the random selection of 300 cells per group on bright field images—selected cells that were not visualized posteriorly on fluorescent fields were considered negative—and fluorescence intensity was compared selecting the whole image field, yielding four pictures of each culture dish. Each culture dish contained recruited leukocytes pooled from the knee joints of 4 mice, and there were 6 pools per group. Analysis was performed by two independent observers blinded to treatments. Fluorescence-positive cells were represented in percentages and fluorescence intensity in arbitrary units of field fluorescence using the same settings for every picture throughout the experiment [67].

### 4.19. TNF-α, IL-1β, and IL-33 Cytokine Levels Determined by ELISA Assay 

We followed the experimental protocol described in Figure 1, part 1. On the third day post-TiO_2_ stimulus, after the mice were euthanized, their knee joints were harvested, and an incision was made to expose the synovia. These explants were cultured in 24-well plates (1 knee joint per well) in 0.5 mL of incomplete RPMI medium in an incubator at 37 °C with 5% CO_2_ for 2.5 h. The conditioned media were collected and frozen for posterior cytokine quantification by ELISA kits for TNF-α (DY410, R&D Systems, Mineapolis, MN, USA), IL-1β (DY401, R&D Systems), and IL-33 (887333, Invitrogen). Briefly, plates were sensitized one day before the experiments with the respective capture antibodies. Frozen media were allowed to thaw slowly on ice while plates were washed, blocked, and standard samples were added. Finally, 0.1 mL of conditioned medium was added, and plates were kept in a state of slow orbital agitation for 2 h at room temperature before carrying out the final washing, detection, substrate addition, and spectrophotometer-reading steps. Plates were read using a microplate spectrophotometer reader (Multiskan GO, Thermo Scientific, Vantaa, Finland) at 450 and 570 nm wavelengths, and the optical density obtained by the 570 nm measurement was subtracted from 450 nm for correction. Results are expressed in picograms of cytokines per milliliter of medium.

### 4.20. Calcium Imaging 

Calcium imaging of DRG neurons was performed as previously described [69]. DRGs were dissected in Neurobasal-A medium (Life Technologies, Thermo Fisher Scientific, Waltham, MA, USA) and dissociated into collagenase A (1 mg/mL)/dispase II (2.4 U/mL; RocheApplied Sciences, Indianapolis, IN, USA) in HEPES-buffered saline (MilliporeSigma, Burlington, MA, USA) for 70 min at 37 °C. After trituration with decreasing sizes of Pasteur glass pipettes, the DRG cells were centrifuged over a 10% BSA gradient and plated on laminin-coated cell culture plates. The DRGs were then loaded with 1.2 μM of Fluo-4AM in Neurobasal-A medium, incubated for 30 min at 37 °C, washed with HBSS, and photographed under a confocal microscope (TCS SP8, Leica Microsystems). To assess TRPV1 activation, DRG plates were recorded for 6 min, which was divided into a 2 min initial reading (0 s mark, baseline values), followed by capsaicin stimulation for 2 min at the 120 s mark (1 μM, TRPV1 agonist [27]) and KCl for 2 min at the 240 s mark (40 mM, activating all neurons). To assess TRPA1 activation, DRG plates were recorded for 6 min, which was divided into 2 min of initial reading (0 s mark, baseline values), followed by stimulation with AITC for 2 min at the 120 s mark and KCl for 2 min at the 240 s mark (40 mM, activating all neurons). Calcium flux was analyzed from the mean fluorescence measured with LAS X software (Leica Microsystems).

### 4.21. Statistical Analysis 

Results are presented as means ± SEM of measurements made for six mice or six pools of samples (each pool derived from 4 mice for ROS and GSH probes and from 6 mice for calcium) in each group per experiment and are representative of two separate experiments. Two-way analysis of variance (ANOVA) followed by Tukey’s post hoc test were used to compare all groups and doses at all times when responses were measured at different times after the stimulus was injected. Differences between responses were evaluated by one-way ANOVA followed by Tukey’s post hoc test for data regarding single time points. In the case of the histopathological score, results were presented as median + range with *n* = 12 animals per group, and statistical analyses were performed using the Kruskal–Wallis test followed by Dunn’s test. Statistical differences were considered significant when *p* < 0.05.

## 5. Conclusions

HMC is a semi-synthetic flavonoid that is clinically used as part of a commercial preparation to treat vascular disease. The present study demonstrates that HMC treatment reduces TiO_2_-induced arthritis pain and the levels of various disease parameters. Its activity also involves reducing the parameters of tissue degradation, such as the level of proteoglycans. These effects were observed in the context of reducing oxidative stress; however, other prior mechanisms that have been shown for HMC might also explain its activity in TiO_2_-induced arthritis. We further advanced the understanding of TiO_2_-induced arthritis’s physiopathology and HMC’s activity by demonstrating that HMC reduces the activation of TRPV1^+^ and TRPA1^+^ nociceptive neurons, which are already activated in basal conditions in TiO_2_-induced arthritis and present a boosted response to their standard agonists, and that HMC reduces such neuronal activation. At the pharmacologically active dose, HMC does not cause side effects that are common to non-steroidal anti-inflammatory drugs. Thus, HMC demonstrates potential to be used as a treatment for prolonged articular inflammation.

## Data Availability

Data are available upon reasonable request made to the corresponding author.

## Sample Availability

This is not applicable since all reagents and compounds used are commercially available.
